# Postpartum Depression and Health: Role of Perceived Social Support among Pakistani Women

**DOI:** 10.3390/diseases11020053

**Published:** 2023-03-31

**Authors:** Samrah Jamshaid, Najma Iqbal Malik, Irfan Ullah, Sundas Saboor, Fauzia Arain, Domenico De Berardis

**Affiliations:** 1 School of Psychology, Northeast Normal University, Changchun 130024, China; jiam093@nenu.edu.cn; 2 Department of Psychology, University of Sargodha, Sargodha 40100, Pakistan; najmamalik@gmail.com; 3 Kabir Medical College, Gandhara University, Peshawar 25120, Pakistan; irfanullahecp2@gmail.com; 4 Institute of Public Health and Social Science (IPH&SS), Khyber Medical University, Peshawar 25120, Pakistan; 5 Khyber Medical College, Peshawar 25120, Pakistan; sundassaboor@hsph.harvard.edu; 6 Icahn School of Medicine, Mount Sinai BronxCare Hospital, New York, NY 10016, USA; fauziaarain@yahoo.com; 7 National Health Service, Department of Mental Health, Psychiatric Service for Diagnosis and Treatment, Hospital “G. Mazzini”, 64100 Teramo, Italy

**Keywords:** insomnia, mental health, physical health, perceived social support, postpartum depression

## Abstract

Postpartum depression (PPD) can predispose to physical and mental health problems in Pakistani women. However, PPD is associated with health and perceived social support, yet their causal relationship is unclear. Therefore, this study intended to evaluate the association of PPD with insomnia, mental health, and physical health. The convenience sampling technique was used to collect data from 320 (52.8%) young and middle-aged postpartum women, at the outpatient departments of obstetrics and gynecology of the Government Maula Bakhsh Hospital, District Head Quarter in Sargodha, Pakistan. The Edinburgh Postnatal Depression Scale, Pittsburgh Sleep Quality Index, Warwick–Edinburgh Mental Well-being Scale, patient health questionnaire, and Multidimensional Scale of Perceived Social Support were used to measure study variables. The results revealed a significant positive relationship between PPD and physical health (r = 0.45, *p* = 0.001), negative relationships with insomnia (r = −0.24, *p* < 0.001), and perceived social support (r = −0.38, *p* = 0.001). Results further confirmed that perceived social support played a moderating role (*β* = 0.97, *p* = 0.01) in the relationship between PPD and mental health among Pakistani women. This study concluded that perceived social support has an important role in PPD and the health of Pakistani women. The study also concluded that poor health is a risk indicator for the identification of aid in the early stages of postpartum among Pakistani women.

## 1. Introduction

Postpartum depression (PPD) refers to a mood disorder, a mixture of complex behavioral, physical, and emotional changes that happen in women after childbirth [1], alongside chemical change [2]. Perceived social support refers to the perception of being cared for by family and friends in a moment of crisis or whenever it is needed [3,4]. Empirical evidence yielded that Pakistani women are at risk of PPD due to familial and cultural factors, such as a lack of awareness about providing special care, including social and moral support to new mothers, while ‘unlucky’ new mothers also tend to be reluctant to seek professional help to deal with postpartum depression because of a lack of awareness [5,6]. Cumbersome activities also increase the pressure on them and, consequently, increase sleep and mental health disorders among postpartum patients who visit clinics [7,8,9]. 

Previous research has demonstrated that depressive symptoms and insomnia correlated with each other before and after the delivery [10]. Furthermore, research has found that women tend to sleep less after birth compared to during their pregnancy and some women also report that they spend more time awake. However, their efficiency to fall asleep decreased from 84 percent to 75 percent after delivery [11,12]. On the contrary, a previous study also explored whether postpartum symptoms correlated to the quality of sleep among women who gave birth in the last three months [13]. Insomnia and sleep problems were highly correlated with each other and caused symptoms of depression during and after pregnancy. Moreover, insomnia and depression both caused negative effects on a mother’s health [14,15,16,17]. Sleep is related to social support, physical health, and mental health [18]. Consequently, a previous study revealed that postpartum depression causes further depression, physical health problems, and mental health problems later in mothers [19,20,21]. 

Perceived social support plays a vital role in predicting and moderating self-related negative life events and depressive symptoms. Furthermore, a study found an interaction effect between perceived social support and negative life events in a clinical group [22], where depression was one of the reasons causing postpartum depression during pregnancy [23]. Perceived social support is found to be a moderator in the relationship between general awareness of psychological symptoms (i.e., OCD symptomology, general psychological symptoms, and depressive symptoms) and the locus of control [24,25]. However, another study determined a novel contribution by psychosocial and biological bodies to explore how neuroticism, perceived social support from the father, and cortisol reactivity contribute to increasing the level of depression during pregnancy [26]. Moreover, multiple studies determined that prenatal depression is at risk and is complicated by psychosocial (i.e., social support and stress, relationship quality) and biological factors (e.g., inflammatory, endocrine, and genetic) [27]. The study conducted by Kwok, Yeung and Chung [28] reported that perceived peer support and physical functional disability are true predictors of depressive symptoms in elders. However, perceived social support is a non-significant predictor when stratified for education level and gender.

The current study aimed to investigate how postpartum depression affects the mental and physical health of Pakistani women by exploring the role of social support in the period of postpartum depression. This research has highlighted some gaps in the literature that need to be addressed in future research to develop optimal evidence-based policy decisions and service provision. The uniqueness of the study is to explore the mental health of females who suffered from postpartum depression and to examine their physical health as well. On the basis of the above literature the following hypotheses were generated to test:

1. There would be a relationship between postpartum depression, insomnia, mental health, physical health, and perceived social support among Pakistani women.
2. Postpartum depression would be a significant predictor of insomnia, mental health, physical health, and perceived social support.
3. Social support would be a significant predictor of postpartum depression, mental health, and physical health.
4. There would be a moderating role of social support in the relationship between postpartum depression and mental health.
5. Employed women who are uneducated would have more postpartum depression compared to educated unemployed women.

## 2. Methods

### 2.1. Materials and Procedures

A cross-sectional research approach was used for the present study. A purposive sampling technique was used, and the samples were recruited between March 2019 and May 2019 from the gynecology and obstetrics outpatient departments, where the mothers visited gynecologists after childbirth and during their postpartum phase. The women who could not read were assisted by the researcher, who read out all the questions and marked all their responses. Data were collected from 385 participants, although after screening them for missing values, outliers, and random responses, only 320 were selected for data analysis.

The eligibility criteria were women who had been in the postpartum phase in the first two to four weeks following childbirth. The terms puerperal period, puerperium, or the immediate postpartum phases are commonly referred to as the first six weeks following childbirth [29]. All the women were aged 16 and above and had at least one baby. In Pakistan, the legal age of adulthood where a male can get married is about 18 years old, while it is 16 years old for a female to get married [30]. However, women younger than 16 years and those with mental health disorders other than PPD were also excluded.

### 2.2. Ethics

This study was approved by the Research Committee of the Department of Psychology, University of Sargodha, Pakistan (SU/PSY/669-S, dated: 09-01-2019). Before data collection, permission was obtained from the Department of Psychology, University of Sargodha, and from the hospital’s medical superintendent (MS) to conduct this research. Procedures and the purpose of the study were introduced and informed consent was obtained from participants. Anonymity and confidentiality were ensured for data responses. This study strictly followed the ethical guidelines of the American Psychological Association (APA).

### 2.3. Measures

The variables of postpartum depression, insomnia, mental health, somatic problems, and perceived social support were measured using the Edinburgh Postnatal Depression Scale (EPDS), Pittsburgh Sleep Quality Index (PSQI), Warwick–Edinburgh Mental Wellbeing Scale (WEMWBS), patient health questionnaire (PHQ-9) and Multidimensional Scale of Perceived Social Support (MSPSS).

Edinburgh Postnatal Depression Scale (EPDS): Previously developed by Cox et al. [31], the EPDS was used in this study to measure postpartum depression in women. This scale consists of 10 items, where every item analyzes a specific function, such as anhedonia, self-blame, anxiety, fear, coping inability, sleeping, sadness, tearfulness, and self-harm [32]. Every item on this scale has a different response format that is scored from 0 to 3, while cumulatively adding the item’s score for each question makes a total that ranges between 0 and 30. The anchor of the scale is different in each question. The alpha reliability of this scale is 0.92. A high value on this scale shows the signs of postpartum depression, while a low value shows no sign of depression in any way.

Pittsburgh Sleep Quality Index (PSQI): The quality of sleep was measured by the PSQI, which was developed by some researchers at the University of Pittsburgh [33]. This scale consists of 15 items of multiple-choice questions. These items measure the seven different components of sleep, which include sleep latency, subjective sleep quality, habitual sleep efficiency, sleep duration, use of sleep medication, sleep disturbance, and daytime dysfunction. Scoring ranges from 0 (no difficulty) to 3 (severe difficulty) and adding the item’s score for each question provides a total that ranges from 0 to 21. The reliability of this scale is 0.56. On this scale, a higher score shows more sleep disturbance, and a low score indicates low disturbance.

Warwick–Edinburgh Mental Wellbeing Scale: To measure the mental health of women, the Warwick–Edinburgh Mental Wellbeing Scale (WEMWBS) was used in the present study. This scale was developed by Stewart and his colleagues [34] and was designed to provide information about mental well-being, including cognitive evaluation dimensions, affective emotional aspects, and psychological functioning. This scale consists of 14 items. The format of each response was a five points Likert scale and the score ranges from 1 to 5. The reliability for this scale is 0.79. A high value on the scale shows positive a high mental health.

Patient health questionnaire: The physical health of the women was measured by the patient health questionnaire, which was developed by Kroenke and his colleagues [35]. This scale is based on a 9-item questionnaire and the scores range from 0 to 3, while the total score ranges from 0 to 27. The reliability for this scale is 0.59. A high value on this measure shows severe physical ailment, while a lower rating shows mild physical ailment.

Multidimensional Scale of Perceived Social Support (MSPSS): Perceived social support was measured by the Multidimensional Scale of Perceived Social Support, which was developed by Zimet, Dahlem, Zimet, and Farley [4]. MPSS consists of 12 items on a 7-point Likert scale that ranges from 1 to 7. This scale has 3 subscales with 4 items for each subscale, which indicate the support of friends, family, and significant others. However, 4 items (1, 2, 5, and 10) assess the support of significant others, 4 items (3, 4, 8, and 11) assess the support of family, and 4 items (6, 7, 9, and 12) assess the support of friends. The reliability of the total scale is 0.71. A high value on this scale shows high social support and a low value indicates a lack of social support.

### 2.4. Statistical Analysis

Following data collection, descriptive statistics were used to show the frequency, percentage, mean, and standard deviation of the study’s variables. Cronbach’s alpha analysis was accessed to find the internal consistency of the study’s instruments. Pearson correlation analysis was performed to explore the relationship between the variables, linear regression analysis accessed to analyze postpartum depression, while perceived social support was used as a predictor of somatic health, mental health, and insomnia. Hierarchal regression analysis was performed to show that perceived social support has a moderating role between postpartum depression and mental health, and two-way ANOVA analysis was performed to show the main effect of education on postpartum depression. The hypotheses of the study were tested with statistical software, IBM SPSS (version 24), and the alpha value was fixed at the 0.05 significance level.

## 3. Results

Table 1 shows the percentage and frequency of demographic factors i.e., age, education, delivery method, newborn gender, employment, family system, number of children, number of daughters and sons, and socioeconomic status. The table shows that the education level of women was low (39.4%) and that they lived in a nuclear family system (78.8%). It also shows that most women had a C-section delivery (64.7%). Furthermore, the results show that most women had 0 to 4 children (83.4%). Most of the participants were from low economic status. In this study, the criteria for measuring social status were set by the monthly income of the family. It is frequently viewed as a combination of economic, social, and occupational status, each of which can be assessed by income, level of education, and type of employment [36].

The results of Table 2 show the mean, standard deviation, alpha reliabilities, and correlation among the variables of the study. The alpha reliabilities coefficient of the overall scales along the subscales ranged from 0.29 to 0.92. Correlation analysis showed that postpartum depression had a significant positive relationship with physical health (*r* = 0.45, *p* < 0.000), while a negative relationship with insomnia (*r* = −0.24, *p* < 0.001), and perceived social support (*r* = −0.38, *p* = 0.001). Furthermore, perceived social support was found to be significantly correlated with the subconstructs, such as with family (*r* = 0.69, *p* < 0.000), friends (*r* = 0.78, *p* < 0.000), and significant others (*r* = 0.83, *p* < 0.000).

**Figure 1:**
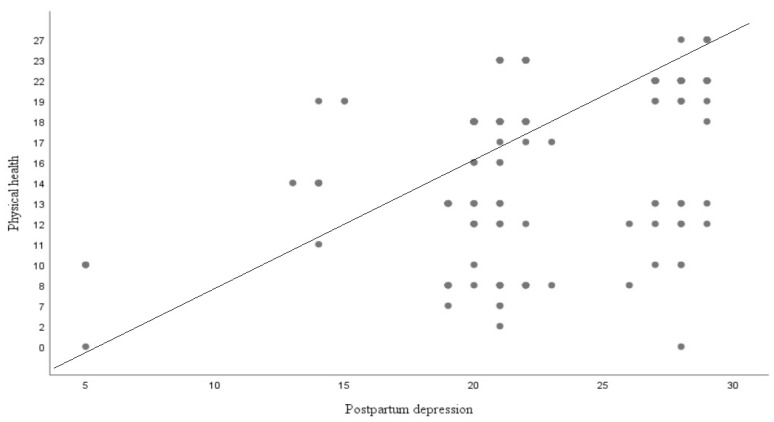
Relationship between study variables.

Figure 1 is scatterplot that demonstrate graphic display for the linear relationship between postpartum depression and physical health.

The result indicates the predictive capability of postpartum depression for insomnia, physical health, mental health, and perceived social support. An *R*^2^ value of 0.06 indicates a 6% variation in the dependent variable, which might account for postpartum depression (*F* (2, 318) = 13.82). These findings indicate that postpartum depression can be a significant predictor of insomnia (*R*^2^ = 0.06, *p* = 0.000, and β = −0.24). The results also revealed significant information on physical health, an *R*^2^ value of 0.45 indicates a 45% variation in the dependent variable, which might account for postpartum depression (*F* (2, 318) = 56.73). The above findings show that postpartum depression is an important predictor of physical health (*R*^2^ = 0.45, *p* = 0.000, and β = 0.21). However, postpartum depression is a non-significant predictor of mental health. Moreover, postpartum depression has been a significant predictor of social support. An *R*^2^ value of 0.14 indicates a 14% variation in the dependent variable, which might be accounted for by postpartum depression (*F* (2, 318) = 36.81). These findings indicate that postpartum depression can be a significant predictor of perceived social support (*R*^2^ = 0.14, *p* = 0.000, and β = −0.38).

The result shows the predictive capability of perceived social support with their subconstructs for postpartum depression, insomnia, physical health, and mental health. The results indicate that perceived social support can be a significant predictor of postpartum depression (*R*^2^ = 0.14, *p* = 0.000, and β = −0.38). An *R*^2^ value of 0.14 shows a 14% variation in postpartum depression, which might be accounted for by perceived social support (*F* (2, 318) = 36.81). The results also indicate that social support can be a significant predictor for insomnia (*R*^2^ = 0.02, *p* = 0.012, and β = 0.16). An *R*^2^ value of 0.02 indicates that there was only a 2% variation in the dependent variable, which might be accounted for by perceived social support (*F* (2, 318) = 6.41). However, perceived social support can be a non-significant predictor of mental health. Furthermore, the results show that social support can be a significant predictor of physical health (*R*^2^ = 0.05, *p* = 0.000, and β = −0.23). An *R*^2^ value of 0.05 shows that there is a 5% variation in physical health, which might be accounted for by perceived social support (*F* (2, 318) = 12.67). However, the results show that the subconstructs were also significant predictors for study variables. 

Table 5 demonstrates the moderating effect of perceived social support (PSS) in the relationship between postpartum depression (PPD) and mental health. Overall model 1 was found to be non-significant (∆*R*^2^ = 0.00, *f* (1,318) = 0.26, *p* = n.s.) because postpartum depression contributed 0% variance to the dependent variable (*R*^2^ = 0.00, 0.607, β = −0.03). Overall model 2 was also found to be non-significant (∆*R*^2^ = 0.00, *f* (1,317) = 0.13, *p* = n.s.) because postpartum depression contributed 0% variance to the dependent variable (*R*^2^ = 0.00, 0.641, β = 00). However, overall, model 3 revealed a significant (∆*R*^2^ = 0.03, *f* (1,316) = 2.67, *p* < 0.05) interaction between postpartum depression and perceived social support, which contributed 3% variance to the dependent variable (*R*^2^ = 0.03, *p* = 0.006, β = 0.97).

**Figure 2:**
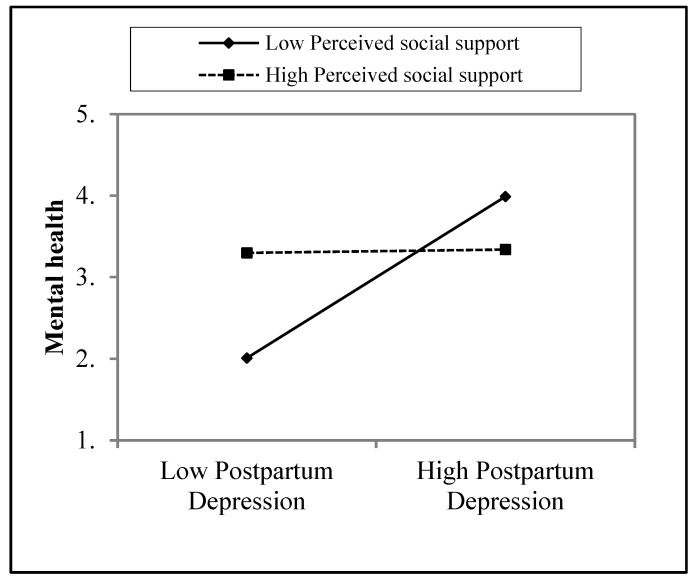
Moderating role of perceived social support in the relationship between postpartum depression and mental health.

The crossed lines on the graph suggest that there is an interaction effect, which is significant for perceived social support. The graph shows that the level of perceived social support is higher for women whose mental health was increased by postpartum depression (Figure 2).

Table 6 shows the main effect of education on postpartum depression, which was found to be significant (*F* (2, 317) = 15.34, *p* < 0.001), while, the main effect of employment on postpartum depression was also found to be significant (*F* (2, 317) = 4.64, *p* < 0.05). Moreover, the interaction between education and employment was found to be significant (*F* (2, 317) = 10.04, *p* < 0.001). The results also show that women who were educated (primary (*M* = 23.90) and middle (*M* = 23.02)) and unemployed (*M* = 22.99) experienced more postpartum depression compared to uneducated women (illiterate (*M* = 20.07)) who were employed (*M* = 21.67).

**Figure 3:**
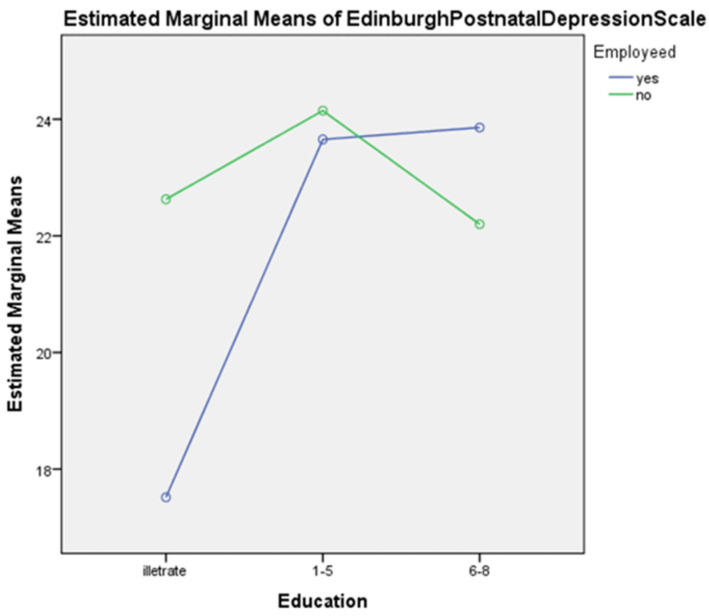
Graphical display of the interacting effect of education and employment on postpartum depression.

Figure 3 depicts that illiterate with high unemployment demonstrate high postnatal depression whereas highly qualified employee individuals display high postnatal depression in comparison to those unemployed. 

## 4. Discussion

This study investigated relationships among the aforementioned study variables and identified significant results, which show that our hypotheses were accepted (see Table 2). The results show that postpartum depression was negatively related to insomnia, mental health, physical health and perceived social support, which indicates that women suffering from higher levels of postpartum depression perceived less social support, which affected their sleep and mental and physical health. The reason for this can be that when women faced more postpartum depression they were more in need of a consultant (i.e., a psychologist) rather than social support, to help them to maintain their health. Another reason can be that usually at a postpartum phase in labor, women have more family members around, however, they perceived more support from their blood relatives than their in-laws. Data were collected from women belonging to rural areas, with a low level of education; however, these women were shy and were unable to share their symptoms of postpartum depression. However, those women whose newborn child was female felt more depressed as the Pakistani culture shows gender discrimination against baby girls. Therefore, this could also be the reason that females do not perceive social support in the postpartum phase, which ultimately affects the health of the women.

This hypothesis was supported by some previous studies. The previous study investigated whether insomnia was stable in women during their pregnancy until two years after the postpartum period using three conditions i.e., feeling stable low, stable medium, and, stable high [16]. A recent study also found a relationship between postpartum symptoms and quality of sleep among women who gave birth in the last three months [13]. Another study [24] has determined that postpartum depression is negatively related to perceived social support among women in Pakistan. A recent study found a negative relationship between social support and postpartum depression. The study showed that they perceived social support so that they can take care of their babies since women are at a high (34.5%) risk for having postpartum depression [37]. Another study also determined that social support, mental health, and health-related quality of life have a relationship with each other [18]. Another study investigated whether postpartum depression predisposed women to develop physical health issues, mental health problems, and chronic depression later in life, conditions that need to be identified early in order to avoid health problems and provide social support [19].

The second hypothesis of the current study was to explore the effect of postpartum depression on insomnia, mental health, physical health, and perceived social support. The results illustrate a low but significant level of regression. Our findings suggest that postpartum depression is a significant predictor of insomnia, physical health, and perceived social support, yet not a true predictor of mental health (see Table 3). The latest studies make a novel contribution to accepting this hypothesis. Research by Kofman and his colleagues combined psychosocial and biological bodies to determine whether neuroticism and perceived social support from the father, alongside cortisol reactivity contribute to increasing the level of depression during pregnancy [26]. Another study investigated whether mental health problems and sleep disorders increased the chances of people visiting clinics [7]. Moreover, a study demonstrated that women sleep less after birth than before. However, their sleep capacities reached 75 percent from 85 percent for awakenings at nighttime [11]. 

Another hypothesis was that social support could be a predictor of postpartum depression, mental health, and physical health. The findings indicate that social support is a predictor of postpartum depression, insomnia, and physical health, although it was not a true predictor of mental health (see Table 4). A previous study has supported this hypothesis. A study by Gan and his colleagues discovered that perceived social support has a significant effect on postpartum depression [38,39] and physical activities [40]. However, another study demonstrated that perceived social support has a strong association with the quality of life and mental health of cancer patients [41]. Perceived social support has a significant impact on the quality of life-related, and symptoms of depression in the general population and in survivors of cancer illnesses [42].

The fourth hypothesis of the study was that there would be a moderating role of perceived social support in the relationship between postpartum depression and mental health. The result indicates that perceived social support has a significant moderating role in the relationship between postpartum depression and mental health (see Table 5 and Figure 1). This hypothesis is supported by previous research in which perceived social support was found to be a significant predictor and moderator, working as a stress-buffering variable between depressive symptoms and stressful negative life events. A further study explored whether perceived social support was a moderator in the relationship between perceived social support and negative life events among clinical groups [22]. Another study also found that perceived social support was a moderator in the relationship between common causality awareness (i.e., psychological symptoms and general symptoms) and locus of control [25]. A previous study explored whether perceived social support was a significant moderator, which affected perfectionism in depression [43]. Furthermore, a recent study demonstrated that perceived social support played a moderating role in the relationship between subjective well-being and perceived discrimination among physically disabled people in Israel [44].

The fifth hypothesis of the study was that employed uneducated women would have more postpartum depression than educated unemployed women. The result shows that women who were educated and unemployed have more postpartum depression compared to uneducated women who were employed (see Table 6 and Figure 2). These findings are supported by another study, which investigated whether postpartum depression prevalence was higher among uneducated women compared to women who are educated, they also examined if the prevalence of depression is common in employed women compared to unemployed women [45]. The findings are similar to old studies that explored prevalence ratios in different developed countries, and they found that these different sources are biological and seldom depend on culture, race, diet, education, and other economic and social factors. However, there is no actual evidence for these differences [46].

There were some limitations to this study. The EPDS scale was developed to measure postpartum depression, although it has similarities with other scales of depression. Future researchers should also interview to get more detailed information. Data were collected from the females who were uneducated or had a lower level of education so it was difficult to get data from uneducated women because they cannot read the questionnaire, meaning the researcher asked the questions and wrote the answers from their responses. The total sample was 320, thus, a suggestion for future researchers is that they interview a large sample size to study postpartum depression in uneducated women. In the main finding, we have found low variance in the study variables, therefore, future researchers should work on studying methodology and statistical analysis to maintain the variance from the result. 

The findings of this study may not be generalizable to all Pakistani women because it did not include females on the edge of the reproductive period continuum in the comparison. After all, some women have premature infants, some women were poor, and some have a history of stillbirth, yet all were having other mental health-related diagnoses. Future researchers should work more on the reproductive period and try to get a sample with fewer physical and mental issues. Further studies should also work on the treatment of sleep, and mental and physical problems in women, which will lead to a decrease in the level of depression in the postpartum phase.

## 5. Conclusions

This study concluded that postpartum depression has a positive relationship with physical health, and a negative relationship with insomnia and perceived social support. The findings also concluded that postpartum depression and social support are significant predictors of insomnia, physical health, and perceived social support. Similarly, the results further concluded that perceived social support plays a significant moderating role in the relationship between postpartum depression and mental health among females. Moreover, the findings concluded that there was a higher level of postpartum depression in working women who were uneducated compared to unemployed educated women.

## Figures and Tables

**Table 1:** Percentage and frequency of study variables (N = 320).

Demographic Variables		*f*	*%*
Age	16–25 years	169	52.8
	26–35	151	47.2
Education	Illiterate	102	31.9
	1–5 class	126	39.4
	6–8 class	92	28.8
Delivery type	C-section	207	64.7
	Normal delivery	113	35.3
Newborn gender	Boy	157	49.1
	Girl	163	50.9
Employed	Yes	154	48.1
	No	166	51.9
Family system	Nuclear	252	78.8
	Joint	68	21.3
Number of Children	0–4 children	267	83.4
	5–10 children	53	16.6
Number of Daughters	0 daughter	103	32.2
	1–3 daughters	134	41.9
	4–6 daughters	83	25.9
Number of Sons	0 Son	40	12.5
	1–3 sons	173	54.1
	4–6 sons	107	33.4
Socioeconomic Status	High economic status	70	21.9
	Moderate economic status	124	38.8
	Low economic status	126	39.4

Note: *f* = frequency.

**Table 2:** Mean, standard deviation, alpha reliabilities, and correlation among study variables (N = 320).

Variables	*M*	*SD*	α	1	2	3	4	5	6	7	8
1 PPD	22.56	4.84	0.92	-	−0.24 ***	−0.03	0.45 ***	−0.38 ***	−0.21 ***	−0.34 ***	−31 ***
2 INS	25.4	3.49	0.56		-	−0.08	−0.14 *	0.17 *	0.12	0.09	0.16 *
3 MH	26.2	4.85	0.79			-	−0.04	0.01	0.18 **	−0.02	−0.06
4 PH	22.98	3.3	0.59				-	−0.23 ***	−0.20 **	−0.22 **	−0.15 *
5 PSS	41.17	5.24	0.71					-	0.69 ***	0.78 ***	0.83 ***
6 FM	15.86	1.6	0.29						-	0.51 ***	0.32 ***
7 FR	13.05	2.07	0.49							-	0.38 ***
8 SO	12.86	3.04	0.81								-

Note: PPD = postpartum depression; INS = insomnia; MH = mental health; PH = physical health; PSS = perceived social support; FR = friends; FM = family; SO = significant others. * *p* < 0.05, ** *p* < 0.01, *** *p* < 0.001.

**Table 3:** Multiple linear regression analysis for postpartum depression to predict insomnia, physical health, perceived social support, and mental health (N = 320).

Variable	INS			PH			MH			PSS		
	*β*	*R* ^2^	*F*	*β*	*R* ^2^	*F*	*β*	*R* ^2^	*F*	*β*	*R* ^2^	*F*
PPD	−0.24 ***	0.06	13.82	0.21 ***	0.45	56.73	−0.03	0.00	0.26	−0.38 ***	0.14	36.81

Note: PPD = Edinburgh postnatal depression scale; INS = insomnia; MH = mental health; PH = physical health; PSS = perceived social support. *** *p* < 0.001.

**Table 4:** Multiple regression analysis of social support along subconstructs predicting postpartum depression, insomnia, mental health, and physical health (N = 320).

Predictor Variables	PPD			INS			MH			PH		
	*β*	*R* ^2^	*F*	*β*	*R* ^2^	*F*	*β*	*R* ^2^	*F*	*β*	*R* ^2^	*F*
PST	−0.38 ***	0.14	36.81	0.16 *	0.02	6.41	0.01	0.00	0.04	−0.23 ***	0.05	12.67
FM	−0.21 **	0.04	10.59	0.12	0.01	3.30	0.18 **	0.03	8.04	−0.20 **	0.04	9.04
FR	−0.33 ***	0.11	28.37	0.09	0.01	2.15	−0.08	0.00	0.07	−0.22 **	0.04	11.34
SO	−0.31 ***	0.09	23.03	0.15 *	0.02	5.65	−0.06	0.00	0.84	−0.14 *	0.01	4.78

Note: PPD = postpartum depression; INS = insomnia; MH = mental health; PH = physical health; PST = perceived social support; FR = friends; FM = family; SO = significant others. *** *p* < 0.001, ** *p* < 0.01, * *p* < 0.05.

**Table 5:** The moderating role of perceived social support in the relationship between postpartum depression and mental health (*N* = 320).

Models	*R* ^2^	β	*F*
Model 1	0.00		0.26
(PPD)		−0.03	
Model 2	0.00		0.13
(PPD)		−0.03	
(PSS)		0.00	
Model 3	0.03		2.67
(PPD)		0.99 **	
(PSS)		0.32 *	
(PPD)*(PSS)		−0.97 **	
Total *R*^2^	0.03		

Note: PPD = postpartum depression scale; PSS = perceived social support scale. * *p* < 0.05. ** *p* < 0.01.

**Table 6:** Two-way ANOVA for the interaction and main effect of education and employment on postpartum depression (N = 320).

Sources	*SS*	*MS*	*F*	Partial *η^2^*
Education	598.59	299.30	15.34 ***	0.125
Employment	90.78	90.78	4.64 *	0.021
Education * Employment	396.18	196.09	10.04 ***	0.086

Note. SS = sum of squares; MS = mean square. *** *p* < 0.001. * *p* < 0.05.

## Data Availability

Data related to the study will be available from the authors upon reasonable request.
